# Gleanings from Our Exchanges

**Published:** 1872-09-01

**Authors:** 


### Gleanings from Our Exchanges.

### TRAUMATIC TETANUS SUCCESSFULLY TREATED BY THE BROMIDE OF POTASSIUM.

BY CEPHAS L. BARD, M. D.

F. S., *æt.* fifteen, daughter of a farmer, during the latter part of April, 1872, stepped upon a bone, the vertebra of a hog, and although the wound produced was deep and bled profusely, yet it healed rapidly, and caused but little inconvenience. Five or six days subsequently she complained of pain in her temples, of stiffness of her jaws, and of difficulty in opening her mouth, which symptoms were attributed by her parents to a bad cold, and treated accordingly. Becoming worse, a neighboring physician, Dr. C. W. Thacker, was summoned, and three days later I was called in consultation. I found all the pathognomonic symptoms of traumatic tetanus present; a rigid, contracted condition of the body, which was bathed with a profuse sour perspiration; opisthotonos; spasms at short intervals; aged expression of countenance, with the *risus sardonicus* well marked; temperature of body, 1050; terrible pain in the epigastrium, due to spasm of diaphragm, which, by former writers, was regarded, when present, as the death warrant to the patient; pulse normal, and mind clear. Retention of urine and constipation of the bowels were also among the symptoms present. There was no pain whatever at the site of the wound, which, to all appearances, had kindly healed, the cicatrix and its surroundings being perfectly firm, and but slightly indurated. Prior to my arrival nothing but morphia had been given, but the symptoms became more appalling, and the intervals between the exacerbations were much shorter. Having laid open the wound, situated in the inner plantar region of the foot, I discovered and removed a small spicula of bone, the tip of the spinous process of the vertebra upon which she had stepped, and which was firmly imbedded among the muscles of her foot. The use of stimulants and nourishing articles of diet were ordered; the warm bath; poultices to the wound; and the bromide of potassium, commencing with thirty grains, given every hour till four doses had been taken, when both the quantity and frequency of administration were lessened. A decided amelioration of the patient's condition almost immediately occurred. The spasms became less powerful and less frequent; the opisthotonos and rigidity of the muscles disappeared; the temperature of the body resumed its normal standard, and she slowly but gradually recovered, though the trismus, which was the last symptom to vanish, existed for three weeks later. During this time she continued to use the bromide, the whole quantity taken amounting to three ounces. No bad effects whatever from its protracted use were noticed, and she is now in the enjoyment of the best of health, enhanced, no doubt, by the recollections of the severe ordeal through which she has passed.

During the last few years this salt has been successfully employed by different members of the profession in several cases of tetanus, and its use in my hands only corroborates the testimony of others.

The pathology of tetanus is obscure; some investigators observing no lesions whatever; others have noticed a congested condition of the membrane enveloping the spinal cord, of the neurilemma of the nerves at their origin and at the site of the wound, when the affection is traumatic. Lockhart, Clarke, and Dickinson, who perhaps have pursued their investigations more closely than others, discovered softening and disintegration of the grey matter of the cord, accompanied by extravasations of blood, caused, no doubt, by an abnormal condition of the blood vessels. They concluded that the tetanic spasms are not solely due to these lesions, which exist also in some other disorders of the nerve centers, but to an abnormal, excitable state of the grey matter, induced by the hyperæmic condition of its blood vessels, and also by the constant irritation of the peripheral nerves. Accepting this theory, no agent could be more suitable, in my opinion, than the bromide of potassium, a vascular and nervous sedative, and whose use is chiefly predicated upon its power of diminishing hyperæmia. It is not claimed that it is a specific, but that it deserves to be ranked as one of our most potent agents in the treatment of such a formidable malady, and deserving of the confidence of the profession.—*Western Lancet.*

Rupture of the Bladder.—Dr. Erskine Mason, reports (*N. Y. Med. Journal*, Aug.'72,) a case of rupture of the bladder through the posterior wall, which he treated successfully by laying open the bladder through the perinæum, as in the lateral operation for stone.

To American surgery, says Dr. Mason, belongs the honor of having given to the profession this mode of treatment; and to Dr. William J. Walker, of Boston, belongs the credit of having first put in practice, and I believe that of originating, this plan of treatment.—*Ibid.*

Case of Complete Prolapsus of the Gravid Uterus.—By Dr. M. H. Biggs, Santa Barbara.—There being so few reported cases of complete prolapsus of the gravid uterus, I thought a short description of the following might be found interesting:

Was hurriedly called upon by an old woman one night about 10 P. M., who said her daughter (aged about 20) was suddenly taken sick and seemed to be dying. Without further explanation I hurried to the patient's bedside and found her almost in a state of collapse; cold perspiration all over; pulse small, quick and scarcely perceptible. Administered brandy at once, and after two or three good doses she recovered sufficiently to whisper that "something had dropped out" and "was hanging between her legs." Upon introducing my hand under the bed clothes, it came in contact with a round, hard, pendulous tumor, extending to within about four inches of her knees. It was about six inches in diameter, and was in fact the prolapsed womb hanging by the relaxed and everted vagina, which could be distinctly clasped between the labia externa and fundus uteri; but why so large?

The patient was not in a condition to answer any more questions, and the old lady had left us alone, probably suspecting more of the cause of the trouble than she wished to communicate. Of course the first thing was to replace the organ, and this was accomplished by using steady pressure applied by the hands, smeared with oil, during about an hour, after which time, to my infinite satisfaction, it popped into its place with an almost audible "flop." Then there was a most acceptable period of repose, during which, after I had made her swallow some broth and more brandy, I insisted upon her communicating to me some of the antecedents of the case, she told me that her courses had stopped —she had missed four periods—and that within the last month she had enlarged so much that her mother had noticed the alteration in her form and charged her with being pregnant, which she of course denied. Mother continued to threaten in case she were so, etc., etc. This made her desperate and she conceived the idea of starving herself, thinking in this way to kill the child and thus produce abortion. She had in fact refrained from all food for the last three days, allaying the cravings of hunger by incessantly smoking cigarettes, and feeling her strength to fail on the afternoon of the third day, had made a last attempt to gain her end by lying on her back on the floor and pulling over on to her belly a "metate," which is a slat of stone used by the native Californians for the purpose of grinding corn, and weighs about thirty to forty pounds. This "metate" she kept in motion, with the little strength she had left, by pushing it up with her hands and at the same time contracting the abdominal muscles, then suddenly relaxing these and pressing down the slat with her hands, and in this way, aided by the momentum imparted to the weight, kept up a kind of pounding movement until she "felt something gradually go down" and "press out." Thinking then that she had "started things in the right direction," she got up and managed to keep about as well as possible until night, when "the something" fell out, but to her horror, instead of separating as she expected, remained hanging down between her legs. She went to bed without taking time to undress, covered herself up and had remained there about two hours in the condition I had found her. While giving her broken account of these sad troubles, slight labor pains came on, and in spite of all that could be done to prevent it, she was delivered a little before daylight of a four or five months fœtus, thus ending a most anxious night.

The patient made a good recovery, and in ten days was attending to her usual avocations; has since been married and had healthy children.

There is no point of special interest in this case except the effect that starvation, aided by the free use of tobacco, may be considered to have had in so completely relaxing the muscular system, as to allow a gravid uterus of such size to be so completely dislocated. For the violence that was used, of itself, would scarcely have sufficed.—*West. Lancet.*

Death-Rate in the United States and Europe.—It is a curious fact, one well worth knowing, that the death-rate in Europe is nearly double what it is in the United States, averaging yearly one out of every forty-three inhabitants, while here it is only one out of every eighty-one. Of the leading countries of Europe, France leads in its mortality, the average being one death to thirty-two people; and England appears to be the healthiest, the deaths being one to every forty-six. In the United States there is a wide range of difference. In Arkansas, for instance, the annual deaths are one to every forty-nine. It appears that the Northwestern States average the healthiest, and the Gulf States the sickliest.—*Med. and Surg. Reporter.*

Progressive Pernicious Anæmia.—Prof. Bimer contributes an article on this affection to the *Medizinische Central Zeitung*, and characterizes it as invariably fatal. Within five years he has seen fifteen cases. The patients have a hydræmic appearance, but are not emaciated, the appetite fails, anæmic sounds are distinguishable in the arteries, capillary hemorrhages take place, especially in the retina, disturbance of vision often takes place, fever is present, and the debility is progressive. Fatty degeneration of the heart and other muscles is present after death.—*Med. Record.*

American Text Books and American Teachers.—American text-books on medicine are the best text-books in the world. Why? Because the materials are selected from the widest range of supply, without prejudice or nationality, pro or con. Whilst the rival nations of the old world are each partial to their own and more or less mistrustful of everything foreign, American writers and teachers have no such prejudice against foreign literature, but rather take pride in appropriating all that they can find in the literature of Great Britain, France, Germany, Italy, and elsewhere. For this reason our text-books are more universal than those of other nations, and an American medical education more cosmopolitan and less national. Our writers and teachers look upwards to the old world and acknowledge its authority. They have not yet built up or discovered a literature of their own. What if we are far removed from the ancient, the greatest and the richest fountains of science? Do not the streams all flow hitherward, and bring tribute from all the world? And does not the river increase by the increase of confluents as you recede from the sources? It follows that the literature, the science and the practice of medicine in America take a wider range, with greater freedom from prejudice and authority, than in the old world. The inventive genius of the Yankee is not confined to washing machines, reapers and steam engines. It pervades every calling, and imparts activity, originality and progress to the practice of medicine.—*Med. and Surg. Reporter.*

Penetrating Wounds of the Knee-Joint.—A French surgeon, Dr. Champerons, according to *Le Mouvement Medical*, has written an elaborate memoir containing his observations on twenty cases of penetrating wounds of the knee-joint by small projectiles, treated during the late European war. His conclusions are that such injuries may be cured and sometimes heal with astonishing facility; that in all cases, and especially when they are extensive, involving periosteum that explorations and drainage tubes must be employed with great caution. Twelve years ago, the late Professor Cooper, of San Francisco, advanced views somewhat similar, and his investigations on wounds of the knee-joint have scarcely been improved on by more recent observers.—*Pac. Med. Jour.*

THORACENTESIS.—The discussion at the French Academy, to which we have several times referred, still continues, and many interesting facts have been elicited.

M. Jules Guerin, in a very masterly summary on the discussion up to that point, and indeed of the whole subject, claimed to have introduced the subcutaneous method of operating more than thirty years ago. He has operated on this plan in fifty-two cases without any accident traceable to the procedures. The plan is to puncture the skin at some little distance from the rest of the puncture, drawing it aside, so that afterwards the perforation is completely closed by natural tissues.

M. Behier criticised all the operations, and especially the statements of M. Chassaignac.

M. Richet reviewed the debate in its surgical aspects, and contributed valuable remarks to the subject of pleural abscess.

M. Sedillot called to mind, without entirely approving it, the teaching of Hippocrates concerning empyema, and rebuked those who sought to claim all credit for modern science, and refused to see that our present state is but the result of long continuous progress. The present and the future, he said, are alike based on the past. He considered that all were substantially agreed on the necessity of opening the thorax whenever a purulent collection was proved to be present. It was also necessary to empty the pleural sac, and to employ injections in order to rectify the surface when needful.

M. Barth, president, drew attention to the fact that in 1865 the Academy had discussed thoracentesis, but that at present no speaker had alluded to that debate, which, nevertheless, elucidated many important points.

M. Richet mentioned a case of empyema in which thoracentesis was performed, but no fluid appeared. At the *post mortem* three separate collections of pus were found enclosed by adhesions, between which the trocar had passed.

The debate, up to a certain point, has been summed up in *La France Medicale*, in a leader signed by our *confrere*, Dr. Lapeyrere. He remarks that five operations were discussed. 1. Simple puncture; 2. Subcutaneous puncture as practiced by M. Jules Guerin; 3. Drainage; 4. Incision; 5. Aspiration. He considers M. Richet's clinical researches on the relative merits of these operations of great value. From them it appears that false membranes frequently occur in empyema, and their presence may prevent either bistoury or trocar coming upon the pus. Moreover, free false membranes are sometimes present, and interfere with drainage. Then incision and injections are called for.

*La France Medicale* has also published in full M. Chassaignac's paper, in which the advantages of drainage are strongly insisted on. This operation, we are told, may well replace various other procedures.—*The Doctor.*

NEW PLAN OF EXTRACTING BODIES FROM THE EAR.—Dr. Loewenberg, of Paris, describes a new plan for extracting solid bodies from the ear, as follows: A very small brush is made by rolling and fixing a narrow strip of old linen around a thin wooden handle (a match, for instance), and unraveling its free border to the length of a quarter of an inch. The end of the so-obtained fringe is dipped into a warm and very concentrated solution of glue, applied to the visible part of the foreign body; or, rather, the operator leans it against the body by letting it glide very softly, and without exercising any pressure, over it. Previous to the application the patient seats himself comfortably in an arm-chair or on a sofa, and inclines the head toward the healthy ear. He remains in this posture for three-quarters of an hour to an hour after the introduction of the agglutinated brush. This time past, consolidation is generally accomplished, and the foreign body can be extracted by gentle pulling at the brush.—*Med. Times and Gaz.*

CEREBRO-SPINAL MENINGITIS.—By Dr. E. Tefft, M.D., Topeka, Kansas.—In the August number of the *Herald*, hypodermic injections of morphia are recommended in cerebro-spinal meningitis, with ice to the back of the neck. The injections I have never used, and consequently have no personal experience in their operation. I have treated my last cases as I do acute rheumatism (and I believe this is the true nature of the disease).

I gave large doses of quinine and Dover's powder in the commencement, alternated with iodo-bromide of calcium (Tilden's) accompanied by warm fomentations to the base of the brain and upper portion of spine. Thus far this treatment has been attended with complete success.

I have just treated one of the most unpromising cases I ever saw with the best of results. The child had partial spasms every few minutes; pupils dilated and contracted alternately to the full extent; head thrown back, gasping for breath, screaming out with agony; urine scanty; extremely tender over base of brain and upper portion of spine; cold feet and hands, and pulse frequent, small and wiry.

I had but little hope of success, but treated it as above indicated, and it improved from day to day until it got perfectly well.

I do not know that the iodo-bromide of calcium is any better than other articles of the same class, but as it is the one I have been using in rheumatic affections more than any other, I used it in this case, and like its effects. I saw a report by a doctor in Illinois, who states that he has cured nearly every case by a similar treatment, and from my own experience I do not hesitate to recommend it to others.—*Leav. Med. Herald.*

Officers and Committees of the Illinois State Medical Society, for 1872.—President, D. W. Young, Aurora, Kane Co.; Vice-President, T. D. Washburn, Hillsborough, Montgomery Co.; 2d Vice-President, F. C. Hotz, Chicago; Treasurer, J. H. Hollister, Chicago; Permanent Secretary, T. D. Fitch, Chicago; Asssistant Secretary, R. D. Bradley, Bloomington; place of meeting, Bloomington.

*Committee of Arrangements.*—T. F. Worrell, M. D., Bloomington; D. L. Crist, M. D. Bloomington; J. L. White, M. D., Bloomington; W. A. Elder, M. D., Bloomington.

*Investigating Committee*—O. Everett, M.D. Dixon; S. P. Breed, M. D., Princeton; E. P. Cook, Mendota.

*Standing Committees.*—On Practical Medicine—S. K. Crawford, M. D., Monmouth; D. L. Jewett, M. D., Watseka; H. M. Lyman, M. D., Chicago. Surgery—J. L. White, M. D., Bloomington; J. B. Hamilton, M. D., Kane; H. W. Kendall, M. D., Quincy. On Ophthalmology—E. L. Holmes, M. D., Chicago; J. P. Johnson, M. D., Peoria; F. C. Hotz, M. D., Chicago. The Committee on Nominations respectfully suggest that the subjects of Ophthalmology and Otology be changed from that of Special Committees to Standing Committees. On Otology—S. J. Jones, M. D., Chicago; Chas. Hunt, M. D., Dixon; Thos. Galt, M. D., Rock Island. On Obstetrics—T. D. Fitch, M. D., Chicago; A. Niles, M. D., Quincy; D. S. Jenks, M. D., Plano. Drugs and Medicines—J. H. Hollister, M. D., Chicago; E. P. Cook, M. D., Mendota; J. P. Hamilton, M. D., Peoria. Necrology—G. W. Albin, M. D., Neoga; J. O. Hamilton, M. D., Jerseyville; J. K. Secord, M. D., Elmwood.

*Special Committees.*—Galt. Therap.—D. Prince, M. D., Jacksonville; Hernia—J. H. Reeder, M. D., Lacon; Hygiene—T. F. Worrell, M. D., Bloomington; On the Assistance Necessary and Justifiable in Difficult and Protracted Labors—S. B. Hawley, M. D., Aurora; Stricture of the Urethra—John Murphy, M. D., Peoria; Phthisis Pulmonalis—H. A. Johnson, M. D., Chicago; Phys. Nerv. Syst.—C. W. Earle, M. D., Chicago; Cerebro-Spinal Meningitis—G. W. Jones, M. D., Danville; Locomotor Ataxia—N. S. Davis, M. D., Chicago; Orthopedic Surgery—J. S. Sherman, M. D., Chicago; to prepare and deliver a public address at the next meeting of the Society—Geo. T. Allen, M. D., Springfield.

Magnetic Wells.—Among the humbugs of the day are magnetic wells. They are found in various parts of the country and are increasing rapidly in number. The magnetic quality of the water is ascertained by connecting a quantity of it in a vessel with the earth by means of a soft iron wire. The wire immediately gives signs of the presence of magnetism when tested by the magnetoscope. Such wells are proved to be highly medicinal and a number of wonderful cures are already reported. Unfortunately, however, some one tried the magnetic experiment with other water, and found that the water of wells and springs in general displayed the same phenomenon. He went farther, and tried the empty cup, without any water, and still the magnetoscope told the same story. It is scarcely necessary to add that the effect is due to terrestrial magnetism, and that nearly all iron is more or less magnetic.—*Pac. Med. Jour.*

Alarming Effects of Chloral in Small Doses.—A correspondent of the *Lancet* furnishes the following cases:

A middle-aged, tall man, recently an attendant at a lunatic asylum, had a boil on the buttock. On the 17th of April I lanced it, and at bedtime the man took part (containing nine grains) of a chloral draught. Shortly he became "stone-cold," his teeth were "fixed," and he stared wildly about. A cold perspiration flowed from him, wetting his pillow and sheets. Clothes in abundance were put on the bed, a fire was lit, and he took warm brandy-and-water. He then began to "feel circulation." The next morning, when I saw him, he looked pale and anxious.

An agricultural laborer's wife, aged sixty-eight, with black hair, and a goitre affecting the middle of the neck, suffered from tumultuous and irregular action of heart, and from pyrosis. She could get little or no rest at night. On the 17th of May she took seven grains and a half of chloral at 9.30 P. M. At 10 she was asleep and slept for two hours. Then she awoke with a scream, jumped out of bed, and sat on the edge of it semi-conscious. She recovered in five minutes, and was got back to bed, where she lay quietly for an hour, and then fell asleep again. In two hours more she awoke with much epigastric pain.

The chloral used in both cases was of Lieberich's manufacture.—*N. Y. Med. Jour.*

Erysipelas.—Professor Broca has lately recommended a fresh plan of treatment, which, according to *L'Abielle Medicale*, he has often successfully employed at the commencement of the disease. This plan is to apply a layer of collodion upon the skin above the part attacked. The layer of collodion, which is to be on sound skin, should be from six to eight centimeters wide, and forming a complete circle, separating the healthy skin from that attacked. A slight circular compression is thus produced, and it is rare for the disease to cross this barrier, behind which it speedily fades. The part should be examined once or twice a day, in order at once to repair any fissures, and the collodion should be quite pure, without any oil, which is sometimes added to it.—*The Doctor.*

Treatment of Hæmoptysis by Iron.—The atomized *liquor ferri subsulphatis* is recommended by the editor of *New Remedies* as the most rational and successful method of treating hæmoptysis. It should be first used of the strength of thirty drops to the ounce, and the strength be increased if needed. Generally the iron salt is very well borne, and does not excite coughing. A saturated solution of alum is less efficient than the iron, says the writer quoted, but may be used when the latter excites irritation.

Skin Grafts.—M. Olliver, of Lyons, thinks that the connective tissus and not the epidermis is the active agent in the grafts, and objects to sowing a number of small epidermir grafts. He cuts them from six to eight centimeters in length, and they succeed very well, but it is inconvenient to procure such large grafts. The skin is first frozen before cutting it away. This is a veritable autoplasty, and the healed surface has a more persistent vitality than ordinary cicatrices.—*Ibid.*

Recovery from Psoas Abscess after Iodide of Potassium.—In the *Medical Achives* for Jan. 1872, Dr. C. Du Hadway reports a case of chronic psoas abscess in which the ingestion of two drachms of iodide of potassium was followed by immediate recovery of the appetite and healing of the abscess in ten days.—*Ibid.*

